# Hemophagocytic lymphohistiocytosis secondary to *Pneumocystis jirovecii* pneumonia: a rare case report

**DOI:** 10.3389/fmed.2026.1795567

**Published:** 2026-04-13

**Authors:** Yanqiang Du, Zhangyan Guo, Dan Yao, Yi Wang

**Affiliations:** Department of Pediatric Intensive Care Unit of Xi’an Children’s Hospital, National Children’s Regional Medical Center (Northwest), Children’s Hospital of Xi’an Jiaotong University, Xi’an, China

**Keywords:** case report, hemophagocytic lymphohistiocytosis, pediatrics, *Pneumocystis jirovecii* pneumonia, secondary HLH

## Abstract

Hemophagocytic lymphohistiocytosis (HLH) secondary to *Pneumocystis jirovecii* pneumonia (PJP) is extremely rare in children. We present the case of a 10-year-old girl with a history of idiopathic thrombocytopenic purpura (ITP) on long-term oral prednisone, who was admitted for progressive fever, cough, and dyspnea. Metagenomic next-generation sequencing of blood and bronchoalveolar lavage fluid confirmed PJP. Despite targeted antifungal therapy and respiratory support, she developed persistent high-grade fever, pancytopenia, hyperferritinemia, hypofibrinogenemia, and hemophagocytosis on bone marrow aspirate by day 10, meeting diagnostic criteria for HLH. Genetic testing was declined by the parents. Management included dexamethasone, continuous renal replacement therapy, and plasmapheresis. Unfortunately, her condition deteriorated, and she was discharged upon parental request on day 22, succumbing on the same day. To our knowledge, this is the first reported pediatric case of HLH secondary to PJP in China. This case highlights that in children with PJP—especially those on immunosuppressive therapy—the development of persistent fever and cytopenia should prompt immediate evaluation for secondary HLH to enable timely intervention.

## 1 Introduction

Hemophagocytic syndrome (HPS), also known as hemophagocytic lymphohistiocytosis (HLH), is a genetic or acquired immunoregulatory dysfunction which is caused by abnormal activation, proliferation and secretion of large amounts of inflammatory cytokines produced by lymphocytes, monocytes and macrophages (1, 2). According to the presence or absence of definite HLH related genetic abnormalities, it can be divided into two categories: primary HLH (pHLH) and secondary HLH (sHLH) (3). In particular, Epstein-Barr virus (EBV) infection is the most common cause of sHLH (4). *Pneumocystis jirovecii* (PJ) infection-related HLH is rare (5–8),and no case has been reported in children in China. Here, a case of HLH secondary to *Pneumocystis jirovecii* pneumonia (PJP) in a child admitted to our hospital is reported as follows, to improve the early clinical recognition of such children by pediatricians.

## 2 Case presentation

A 10-year-old girl was admitted to our hospital on May 3, 2022, due to fever and cough for 3 days; respiratory symptoms worsened and dyspnea developed 1 day prior to admission. Fever was present during this period, and body temperature fluctuating between 37.4°C and 38.3°C. Her respiratory symptoms were characterized by dry cough and progressive dyspnea. She was treated with antibiotics in another hospital, but the symptoms did not improve. Therefore, she was transferred to our department.

She was diagnosed with idiopathic thrombocytopenic purpura (ITP) in November 2021 at an another hospital. She was initiated on oral prednisone at a dose of 2 mg/kg/day, which was gradually tapered. At the time of admission to our hospital, she was receiving prednisone 15 mg twice daily.

On admission, the girl was alert, communicative, tachypneic (RR 50/min) with fine basal rales on chest auscultation; and no special conditions in other important organs and systems.

**FIGURE 1:**
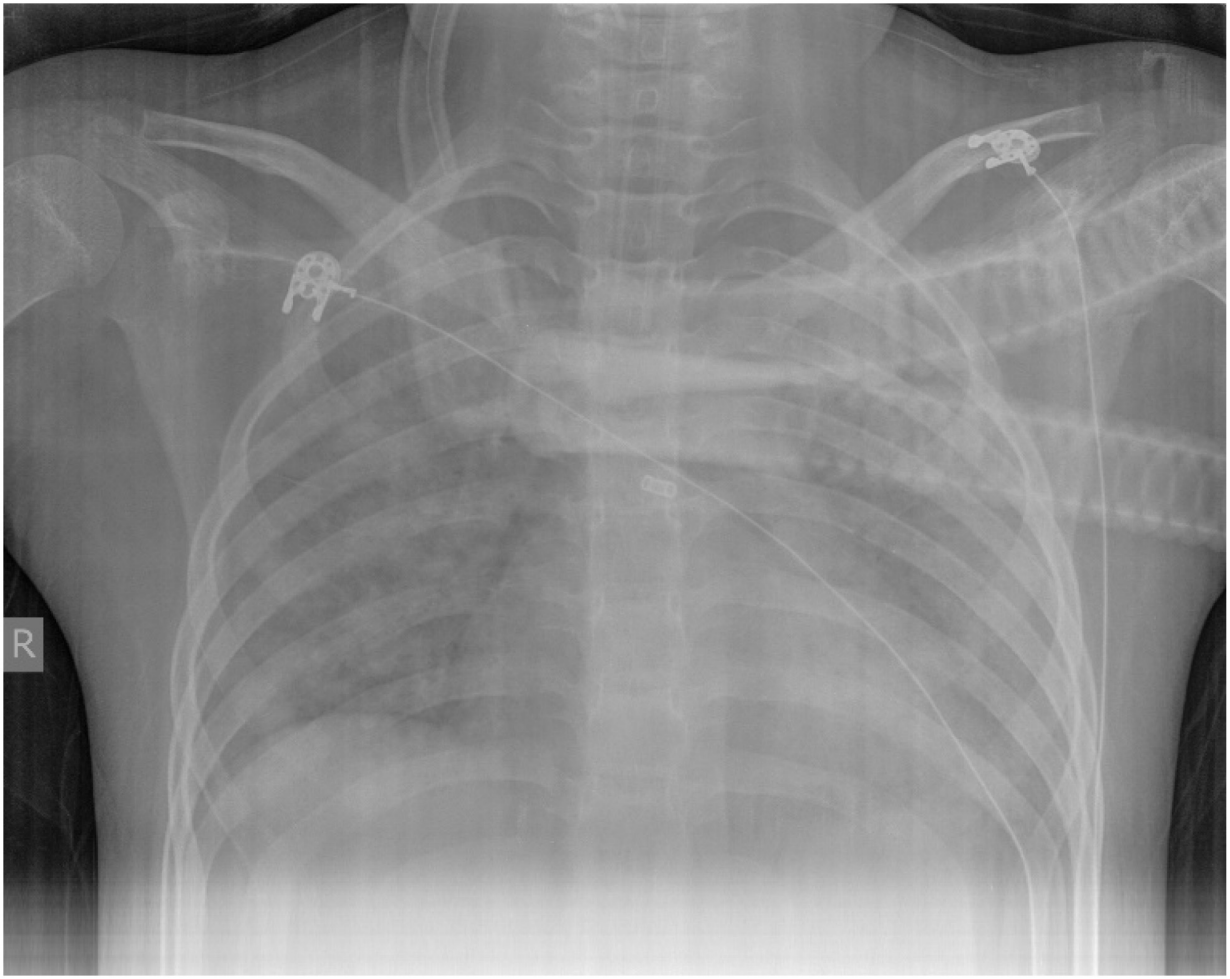
X-ray of the patient. Diffuse exudative lesion in both lungs (day of admission).

Laboratory examination: white blood cell count (WBC) 5.85 × 10^9^/L, neutrophil (NE) 4.92 × 10^9^/L, hemoglobin (HGB) 108 g/L, platelets (PLT) 261 × 10^9^/L, C-reactive protein (CRP) 25.2 mg/L (reference range 0–3), procalcitonin (PCT) 0.27 ng/ml (reference range 0–0.05), plasma (1,3)-β- D-glucan test (BDG) 154.2 pg/ml (reference range 0–60), lactate dehydrogenase (LDH) 1495 U/L (reference range 120–246), serum ferritin level 282.6 ng/ml(reference range 4–204). Gram-negative bacteria lipopolysaccharide was negative. Serology assays for HIV, CMV, HCV, and HBV were negative. Liver and renal function, electrolytes, and myocardial enzymes were normal. The serum levels of immunoglobulin M, G, and A were normal. The CD4^+^ lymphocyte count, B lymphocyte count and NK cell count were normal. Blood gas analysis: PH 7.39,pCO_2_ 26 mmHg,pO_2_ 50 mmHg,Lac 0.7 mmol/L,BE −8 mmol/L,SaO_2_ 88.7%. Chest X-ray indicated diffuse exudative lesion in both lungs (Figure 1).

**FIGURE 2:**
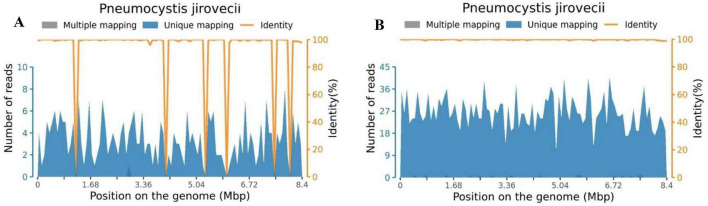
*Pneumocystis jirovecii* genome coverage map of the patient. **(A)** Blood specimen. **(B)** BALF specimen. BALF, bronchoalveolar lavage fluid.

**FIGURE 3:**
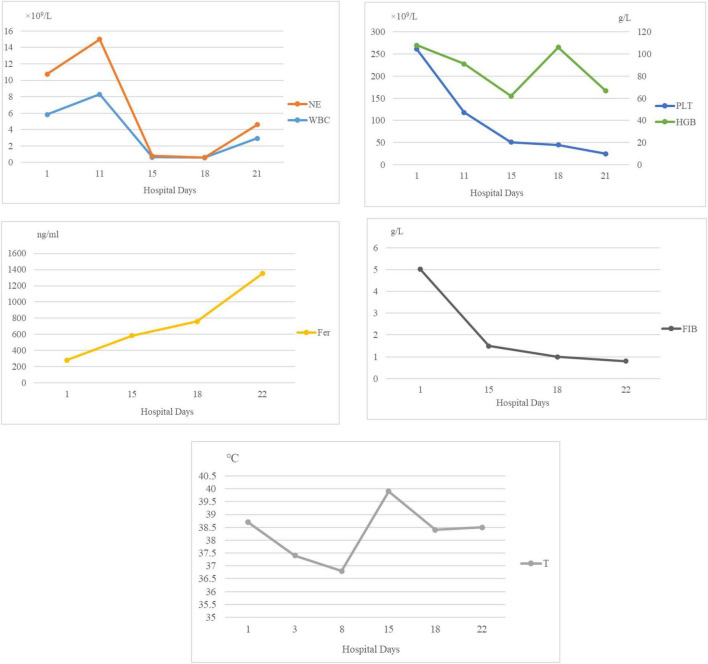
White blood cell count (WBC) and NE decreased to the normal values after antibiotic treatment, and then gradually decreased to below the normal values. HGB continued to decline on admission, rebounded after we administered a blood transfusion, and was slightly low again. PLT and FIB continued to decrease after admission. Fer increased progressively after admission. Body temperature gradually decreased to normal after antibiotic treatment, but on the 8th day of admission, the child’s body temperature gradually increased. WBC, white blood cell; NE, neutrophils; PLT, platelets; HGB, hemoglobin; FIB, fibrinogen; Fer, Ferritin; T, body temperature.

**FIGURE 4:**
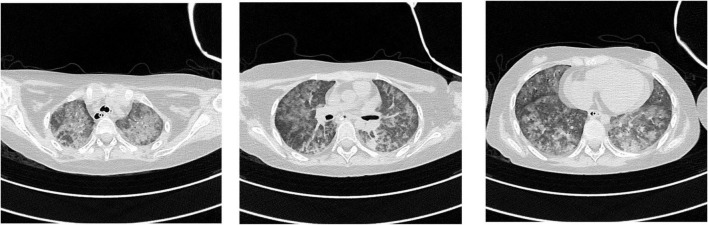
Chest computed tomography (CT) of the patient (10 days after admission). Extensive ground-glass opacities were observed in both lungs.

**FIGURE 5:**
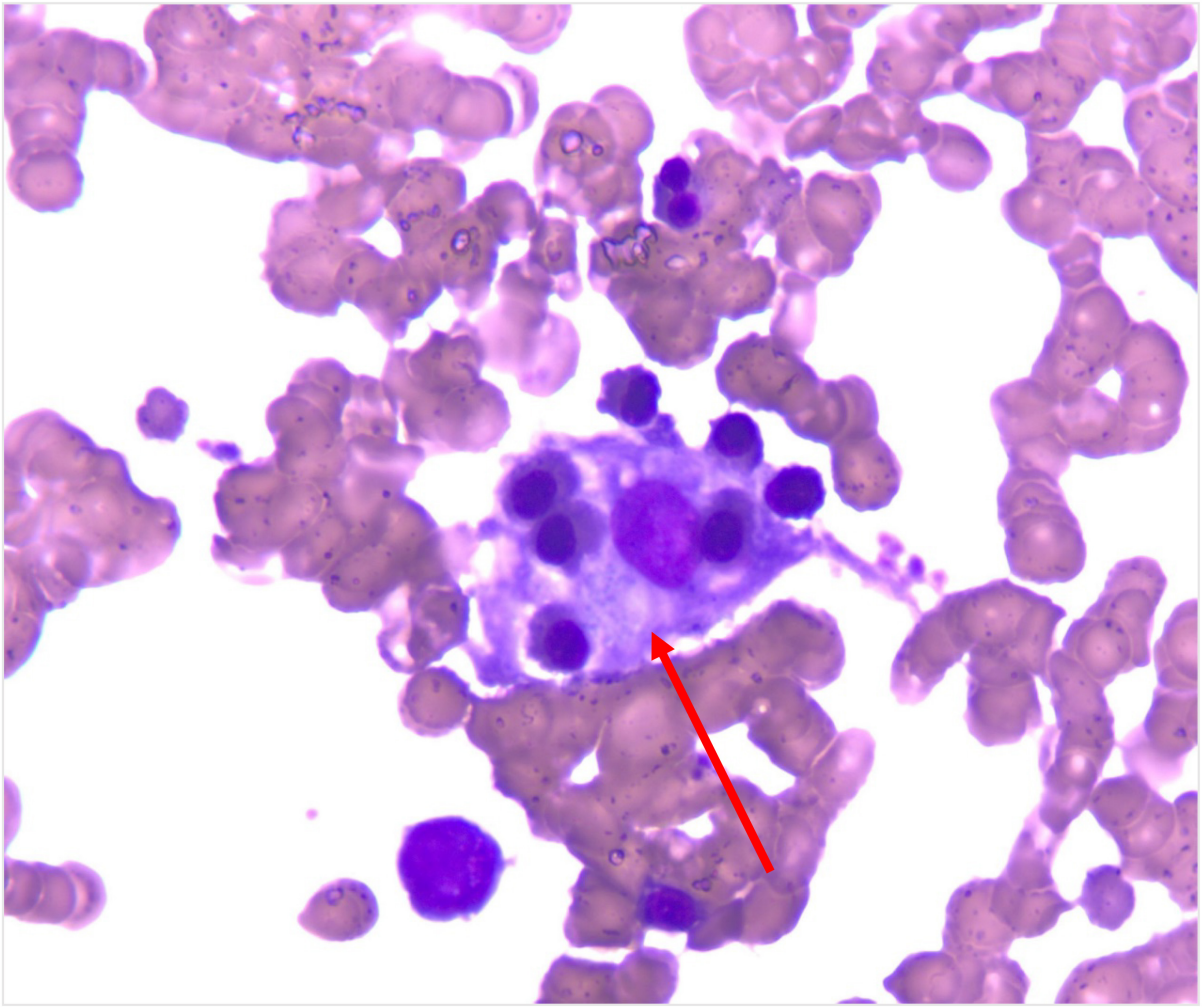
Bone-marrow smear with hemophagocytosis (Wright’s stain, ×100).

After admission, she was intubated and mechanically ventilated. Meropenem was used for anti-infective treatment. On the third day after hospitalization, metagenomic next-generation sequencing (mNGS) of blood detected *Pneumocystis jirovecii* with a specific sequence number of 326 (Figure 2A). mNGS of bronchoalveolar lavage fluid (BALF) also detected *Pneumocystis jirovecii* with the specific sequence number of 2644 (Figure 2B), and the antibiotics were adjusted to caspofungin combined with trimethoprim-sulfamethoxazole (TMP-SMZ). 6 days after admission, the blood and BALF bacterial culture was negative. The girl was treated with prone position ventilation, due to pediatric acute respiratory distress syndrome (pARDS) was considered. Despite above treatments, the child’s respiratory condition did not improve. On the 10th day, her body temperature fluctuated between 38°C and 40°C, laboratory examination showed that WBC, NE, HGB and PLT decreased to 0.65 × 10^9^/L, 0.14 × 10^9^/L, 62 g/L, 51 × 10^9^/L, respectively; serum ferritin level was increased progressively at 1351.76 ng/ml; fibrinogen (FIB) was reduced to 1 g/L; triglycerides increased to 3.85 mmol/L; an NK cell activity assay was 2% (reference range ≥ 4%); solubleinter leukin-2 receptor levels (sCD25) was 2421 U/ml (reference range 223–710) (Figure 3). Due to the laboratory testing limitations of our hospital (the hospital’s clinical laboratory does not have the corresponding detection kits and technical platforms for these two biomarkers), IL-18 and CD163 were not tested. Chest computed tomography (CT) showed extensive ground-glass opacities (GGO) in both lungs (Figure 4). Bone marrow aspirate showed an extensive hemophagocytosis (Figure 5). So she was diagnosed with HLH, but her parents refused to complete genetic investigations. Given the severity of the pulmonary infection, she was treated with dexamethasone in combination with continuous renal replacement treatment (CRRT) and plasmapheresis instead of chemotherapy. Unfortunately, her pulmonary infection was getting worse (Ventilator parameters: FIO_2_ 100%, PEEP 12cmH_2_O, VT 6 ml/kg), and she was discharged on day 22, because her parents gave up treatment. We knew that she died on the day of discharge, through telephone follow-up.

## 3 Discussion

PJP is an opportunistic fungal pneumonia caused by PJ (9), usually prevalent in human immunodeficiency virus (HIV)-infected patients (10),and is the main cause of their death. But with the use of antiretroviral drugs in clinical practice, the incidence and mortality rates of HIV-associated PJP have decreased significantly (11). In recent years, with the increase in the number of patients with autoimmune diseases, hematologic tumors, solid tumors, and organ transplants, biologics, glucocorticoids, and immunosuppressive agents have been widely quoted in the clinic, leading to a gradual increase in patients with acquired immunodeficiency, and a significant increase in the incidence and mortality of non-HIV-related PJP. The girl was diagnosed with ITP half a year ago, and has been treated with oral glucocorticoids for a long time. Therefore, the child is a high-risk group of PJP infection, and clinicians should be alert to the possibility of PJP in such children.

HLH secondary to PJP is rare, and only a few cases have been reported. Koduri et al. reported a case of HLH in a 41-year-old male with HIV-associated PJP in 1995, but the treatment process of this case was not described in detail (8). Karras et al. reported 17 patients with sHLH after kidney transplantation, but only one patient had HLH induced by PJP (6). Similarly, Chinese scholar Xie et al. reported 7 patients with PJP after kidney transplantation, and only one patient developed HLH secondary to PJP (12). Machaczka et al. reported a 74-year-old male with Hodgkin’s lymphoma combined with PJP who developed HLH secondary to PJP (7). However, sHLH can be also developed in patients with Hodgkin’s lymphoma (13), so it could not be explained that this patient developed HLH secondary to PJP. Pasic et al. reported a 10-year-old boy who was admitted to the hospital due to fever, dry cough, and shortness of breath, and was diagnosed with PJP. However, he was also complicated with hepatosplenomegaly, elevated serum ferritin, and decreased peripheral blood hemoglobin and platelets, so HLH was diagnosed (5). But we can’t distinguish whether he had HLH secondary to PJP or PJP secondary to HLH. Henter et al. reported that 1 of 7 children with familial hemophagocytic lymphohistiocytosis (FHL) had PJP as a complication during treatment, which suggested that children with HLH could be infected with PJP (14). In our case, the girl was diagnosed with PJP after admission. Her clinical signs and symptoms were transiently improved during hospitalization, but then a persistent high fever and progressive reduction of three lineage cells occurred, which led to the diagnosis of HLH, finally.

HLH is characterized by intermittent fever, hepatosplenomegaly, pancytopenia, hypertriglyceridemia, and hypofibrinogenemia and, histologically, by a non-malignant lymphohistiocytic infiltration with hemophagocytosis in reticuloendothelial organs (3). When children with PJP have persistent fever and high inflammatory response during the treatment, it is necessary to be alert to the possibility of sHLH, and the relevant examinations of HLH should be performed as soon as possible to make a definite diagnosis. The positive diagnostic indicators of HLH present in this case are as follows: (1) persistent high-grade fever (body temperature fluctuating between 38°C and 40°C on the 10th hospital day); (2) pancytopenia (leukopenia: 0.65 × 10^9^/L, anemia: hemoglobin 62 g/L, thrombocytopenia: 51 × 10^9^/L); (3) hyperferritinemia (serum ferritin 1351.76 ng/ml, far exceeding the diagnostic threshold); (4) hypofibrinogenemia (fibrinogen 1 g/L); (5) elevated soluble interleukin-2 receptor (sCD25: 2421 U/ml, above the reference range); (6) decreased natural killer (NK) cell activity (2%, below the reference range of ≥4%); (7) pathological evidence of hemophagocytosis in bone marrow aspirate; (8) hypertriglyceridemia (triglycerides 3.85 mmol/L). All the above positive indicators fully meet the 2004 diagnostic criteria for sHLH (1). A limitation of our study is that we did not complete the primary immunodeficiency genes and pHLH-related genes of the child. Our patient is 10 years old and in good health with no opportunistic pathogen infection, no history of repeated chronic infection and no history of HIV infection before this hospitalization. Therefore, it is considered that the child has no primary or secondary immunodeficiency, similar to literature reports (15–17). The patient diagnosed with ITP before 6 months and given glucocorticoid therapy, which may be the cause of her infection with PJ and remind clinicians to strengthen the monitoring of opportunistic infections in children receiving long-term immunosuppressive therapy.

Therefore, we believe that this patient is highly likely to develop HLH secondary to PJP.

## 4 Conclusion

To our knowledge, this is the first pediatric case of HLH secondary to PJP reported in China, which enriches the clinical spectrum of pediatric PJP and sHLH in the Chinese population. This case underscores the importance of early consideration of sHLH in children with PJP—particularly those with a history of immunosuppressive therapy—who develop persistent fever and cytopenia during treatment. Prompt screening of core HLH diagnostic indicators (including sCD25, ferritin, NK cell activity, etc.) is essential for early diagnosis and intervention. Multi-center, large-sample clinical studies are needed in the future to further clarify the clinical characteristics and optimal treatment strategies of PJP-associated sHLH in children.

## Data Availability

The raw data supporting the conclusions of this article will be made available by the authors, without undue reservation.
